# Gene expression analysis of flax seed development

**DOI:** 10.1186/1471-2229-11-74

**Published:** 2011-04-29

**Authors:** Prakash Venglat, Daoquan Xiang, Shuqing Qiu, Sandra L Stone, Chabane Tibiche, Dustin Cram, Michelle Alting-Mees, Jacek Nowak, Sylvie Cloutier, Michael Deyholos, Faouzi Bekkaoui, Andrew Sharpe, Edwin Wang, Gordon Rowland, Gopalan Selvaraj, Raju Datla

**Affiliations:** 1 Plant Biotechnology Institute, NRC, 110 Gymnasium Place, Saskatoon, Saskatchewan, S7N 0W9, Canada; 2 Computational Chemistry and Bioinformatics Group, Biotechnology Research Institute, NRC, 6100 Royalmount Avenue, Montreal, Quebec H4P 2R2, Canada; 3 Cereal Research Centre, Agriculture and Agri-Food Canada, Winnipeg, MB, R3T 2M9, Canada; 4 Department of Biological Sciences, University of Alberta, Edmonton, Alberta, T6G 2E9, Canada; 5 Crop Development Centre, University of Saskatchewan, Saskatoon, Saskatchewan, S7N 0W9, Canada

## Abstract

**Background:**

Flax, *Linum usitatissimum *L., is an important crop whose seed oil and stem fiber have multiple industrial applications. Flax seeds are also well-known for their nutritional attributes, viz., omega-3 fatty acids in the oil and lignans and mucilage from the seed coat. In spite of the importance of this crop, there are few molecular resources that can be utilized toward improving seed traits. Here, we describe flax embryo and seed development and generation of comprehensive genomic resources for the flax seed.

**Results:**

We describe a large-scale generation and analysis of expressed sequences in various tissues. Collectively, the 13 libraries we have used provide a broad representation of genes active in developing embryos (globular, heart, torpedo, cotyledon and mature stages) seed coats (globular and torpedo stages) and endosperm (pooled globular to torpedo stages) and genes expressed in flowers, etiolated seedlings, leaves, and stem tissue. A total of 261,272 expressed sequence tags (EST) (GenBank accessions LIBEST_026995 to LIBEST_027011) were generated. These EST libraries included transcription factor genes that are typically expressed at low levels, indicating that the depth is adequate for *in silico *expression analysis. Assembly of the ESTs resulted in 30,640 unigenes and 82% of these could be identified on the basis of homology to known and hypothetical genes from other plants. When compared with fully sequenced plant genomes, the flax unigenes resembled poplar and castor bean more than grape, sorghum, rice or Arabidopsis. Nearly one-fifth of these (5,152) had no homologs in sequences reported for any organism, suggesting that this category represents genes that are likely unique to flax. Digital analyses revealed gene expression dynamics for the biosynthesis of a number of important seed constituents during seed development.

**Conclusions:**

We have developed a foundational database of expressed sequences and collection of plasmid clones that comprise even low-expressed genes such as those encoding transcription factors. This has allowed us to delineate the spatio-temporal aspects of gene expression underlying the biosynthesis of a number of important seed constituents in flax. Flax belongs to a taxonomic group of diverse plants and the large sequence database will allow for evolutionary studies as well.

## Background

Flax (*Linum usitatissimum *L.) is a globally important agricultural crop grown both for its seed oil as well as its stem fiber. Flax seed is used as a food source and has many valuable nutritional qualities. The seed oil also has multiple industrial applications such as in the manufacture of linoleum and paints and in preserving wood and concrete. The fiber from flax stem is highly valued for use in textiles such as linen, specialty paper such as bank notes and in eco-friendly insulations [1]. Flax belongs to the family *Linaceae *and is one of about 200 species in the genus *Linum *[2]. It is a self-pollinating annual diploid plant with 30 chromosomes (2n = 30), and a relatively small genome size for a higher plant, estimated at ~700 Mbp [3,4]. Although flax demonstrates typical dicotyledonous seed development, there are species-specific differences compared to, for instance, *Arabidopsis thaliana *seed development. However, very little is known about genes expressed during flax seed development. Advancing this knowledge and comparison of gene expression profiles and gene sequences would provide new insights into flax seed development.

Nutritionally, flax seed has multiple desirable attributes. It is rich in dietary fiber and has a high content of essential fatty acids, vitamins and minerals. The seeds are composed of ~45% oil, 30% dietary fiber and 25% protein. Around 73% of the fatty acids in flax seed are polyunsaturated. Approximately 50% of the total fatty acids consist of α-linolenic acid (ALA), a precursor for many essential fatty acids of human diet [5]. Flax seed is also a rich source of the lignan component secoisolariciresinol diglucoside (SDG). SDG is present in flax seeds at levels 75 - 800 times greater than any other crops or vegetables currently known [6,7]. In addition to having anti-cancer properties, SDG also has antioxidant and phytoestrogen properties [8]. Flax seed contains about 400 g/kg total dietary fiber. This seed fiber is rich in pentosans and the hull fraction contains 2-7% mucilage [9]. The other major constituent of flax seeds are storage proteins that can range from 10-30% [10]. Globulins are the major storage proteins of flax seed, forming about 58-66% of the total seed protein [11,12].

Improvement of flax varieties through breeding for various traits can be assisted by development of molecular markers and by understanding the genetic and biochemical bases of these characteristics [13,14]. The goal of this research was to develop a comprehensive genomics-based dataset for flax in order to advance the understanding of flax embryo, endosperm and seed coat development. We report the construction of 13 cDNA libraries, each derived from specific flax seed tissue stages, as well as other vegetative tissues together with the generation of ESTs derived from these libraries and the related assembled unigenes. We mined the resulting database with the goal of revealing new insights into the gene expression in developing seeds in comparison to that of vegetative tissues and other plant species. We show the usefulness of this database as a tool to identify putative candidates that play critical roles in biochemically important pathways in the flax seed. Specifically we analyzed gene expression during embryogenesis as related to fatty acid, flavonoid, mucilage, and storage protein synthesis and transcription factors.

## Results and Discussion

### Seed development characteristics in flax

**Figure 1:**
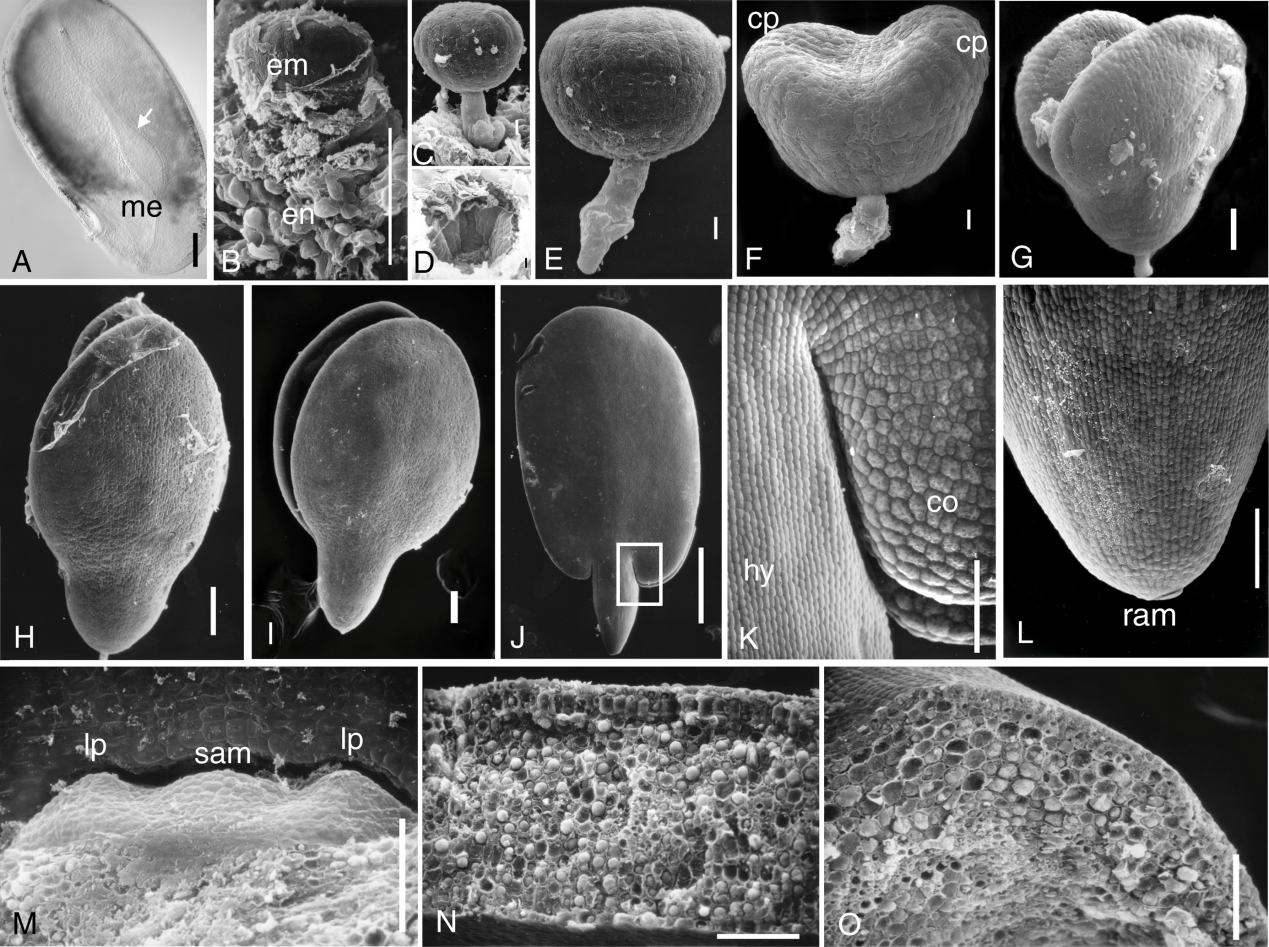
**Flax embryo development**. (A) Cleared seed soon after fertilization. The embryo sac (arrow) encloses the embryo and endosperm and is anchored in the micropylar end (me) of the thick seed coat. (B-O) Scanning electron microscopy of developing flax embryo. (B) Dissected micropylar end of the seed showing endosperm cells (en) surrounding the developing globular embryo (em). (C) Globular embryo with suspensor anchored at the micropylar end. (D) Micropylar sleeve that remains after removal of the globular embryonic suspensor. (E) Globular embryo. (F) Heart embryo. The cotyledon primordia are indicated by "cp". (G) Early torpedo embryo. (H) Late torpedo embryos with pointed cotyledon tips. (I) Cotyledon stage embryo with rounded cotyledon tips. (J) Mature embryo with elongated cotyledons and a short embryonic axis. (K) Higher magnification of the cotyledon (co) and hypocotyl (hy) as indicated by the inset rectangle shown in (J). (L) The radicle tip showing the embryonic root apical meristem (ram). (M) The embryonic shoot apical meristem (sam) and leaf primordia (lp). Mature embryonic (N) cotyledon and (O) hypocotyl in cross-section to show cellular differentiation and storage deposits. Bar = 1 mm (J), 0.1 mm (A, B, G-I, K-O) and 10 μm (C-F).

**Figure 2:**
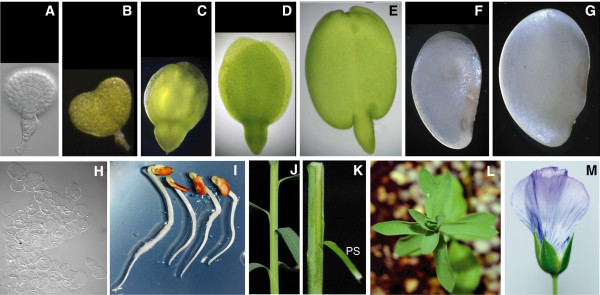
**Flax tissues used for cDNA library construction and EST analysis**. (A) globular embryo; (B) heart embryo; (C) torpedo embryo; (D) cotyledon embryo; (E) mature embryo; (F) globular stage seed coat; (G) torpedo stage seed coat; (H) pooled endosperm from globular to torpedo stage seed; (I) etiolated seedlings; (J) stem; (K) stem peel "PS"; (L) leaves; and (M) mature flower.

Limited information is available regarding flax seed development, despite its economic importance. Since the seed is an economically important output of this crop, in this study, we performed a detailed analysis of embryogenesis and flax seed development. The flax seed consists of three major tissues: the diploid embryo and triploid endosperm as products of double fertilization, and the maternal seed coat tissue. Soon after fertilization, the seed is translucent and the embryo sac is upright within the integuments (Figure 1A). The developing embryo is anchored at the micropylar end of the embryo sac. The thick, clear and fragile integuments of the fertilized ovule differentiate into the thin, dark and protective seed coat during seed development. Observation during the dissection process revealed that the endosperm initials, which formed at fertilization, undergo divisions to form a cellularized endosperm by the globular embryo stage (Figure 1B and Figure 2H). The endosperm progressively increases in size up to the torpedo stage, after which time it begins to degenerate, presumably to make space for the rapidly elongating cotyledons and to provide nutritional support to the developing embryo. By the late cotyledon stage the majority of endosperm cells have been consumed, leaving a thin layer of endosperm on the inner wall of the seed coat of the maturing seed.

The globular embryo (Figure 1C, 1E) has a short suspensor consisting of just four cells that is nestled into the micropylar sleeve (Figure 1D). As the embryo develops from the globular (Figure 1E) to heart (Figure 1F) and torpedo (Figure 1G) stages, the increase in embryo size is largely due to growth of the cotyledons. This is in contrast to the Arabidopsis embryo where the increase in size is due to an increase in both the cotyledons and the embryonic axis [15]. The embryonic axis consists of the hypocotyl and radicle initials that are formed at the heart stage and it eventually differentiates to form a short peg-like structure in the mature embryo. Whereas the tips of the cotyledon primordia are pointed in the late torpedo stages (Figure 1H) they become rounded at the top in the cotyledon stage (Figure 1I). The mature embryo (Figures 1J, 1K) is primarily composed of two large cotyledons, and a relatively short embryonic axis. The cotyledons play a dual role nutritionally during germination and early seedling growth. They hold much of the seed storage reserves and become photosynthetic after germination. The mature embryo contains dormant leaf primordia initials and shoot and root apical meristems that will become activated after imbibition and during the germination of the seed (Figures 1L, 1M). A cross-section of the cotyledon shows differentiation of the cortical cells into a layer of palisade cells and the compact mesophyll cells. The mesophyll cells of the cotyledon and the parenchyma cells of the hypocotyl are filled with storage deposits (Figure 1N, 1O) similar to those previously reported [16]. While flax seed development follows the general trends described for seeds of other model dicot species, there are some features that are different. For instance, unlike the Arabidopsis embryo, where the mature embryo is bent inside the anatropous seed, the flax embryo is positioned upright within the seed [15]. In the flax seed, the cotyledons take up the majority of the seed space with only a thin endosperm and seed coat left at maturity. This is in contrast to castor bean seeds where the endosperm is thick and the cotyledons nestled within the endosperm are thinner [17].

### Sequencing 13 cDNA libraries provides insights into the flax transcriptome

The cDNA libraries constructed in this study provide a broad representation of seed development (8 libraries) as well as 5 libraries for vegetative tissues. The 8 seed libraries were all from the most widely cultivated Canadian linseed variety CDC Bethune and comprised globular embryo, heart embryo, torpedo embryo, cotyledon embryo, mature embryo, seed coat from the globular stage, seed coat from the torpedo stage and pooled endosperm (globular to torpedo stage) (Figure 2 A-H); four of the remaining five cDNA libraries were prepared from whole etiolated seedlings, stem, leaf, and flowers (Figure 2 I, J, L and 2M) of cv. CDC Bethune and the last library was for stem peels from cv. Norlin (Figure 2K).

The EST collection from single pass sequencing of the 3' end of the cDNA in plasmid clones had a median length of 613 nucleotides (nt). Each of these clones has been catalogued and stored at -80°C to allow for further studies. Full length cDNAs have also been identified for some clones by additional 5' end sequencing. Table 1 summarizes the distribution, quantity and quality of the ESTs obtained from the 13 libraries. After removal of vector sequences, rRNA sequences, sequences <80 nt, organelle sequences and masking for repeats, 261,272 sequences remained. The assembly of a final unigene set was done in two steps. First, ESTs from each library were assembled with EGassembler [18], resulting collectively in 27,168 contigs and 51,041 singletons. This collection of 78,209 contigs and singletons was reassembled with EGassembler. Thus a unigene set for each tissue source and a unified set of unigenes encompassing all the tissues were obtained. This second assembly process resulted in 15,784 contigs and 14,856 singletons, totaling 30,640 unigenes. The 30,640 unigenes identified here likely represents a major part of the flax seed transcriptome. Table 2 shows the distribution of the clusters, contigs, singletons and unigenes in the individual libraries. The length of the contigs varies from 102 to 3,027 nucleotides with a median length of 778 nt (data not shown). The sum of the lengths of the contigs plus singletons is 21.6 megabases, which represents 3% of the predicted 700Mb flax genome [3]. The EST distribution for each unigene among the 13 tissues and its predicted or putative Arabidopsis homologue is presented in Additional File 1. A queryable flax unigene database is available at http://bioinfo.pbi.nrc.ca/portal/flax/ and all the EST sequences are also deposited in GenBank (Table 3). Of the 30,640 unigenes, 23,418 (76.4%) were identified as having significant homology with Arabidopsis gene sequences. The Arabidopsis genome is ~157 Mbp [19] and has a transcriptome of ~27,000 genes [20] and our analysis hints that flax potentially has a larger transcriptome than Arabidopsis. While our libraries do not give complete coverage of the flax vegetative tissues, they can be used as minimum number to estimate the size of flax transcriptome.

**Table 1:** Distribution and analysis of flax ESTs in the 13 libraries

Tissue library	Number of ESTs sequenced	Number after cleaning	Number masked	% Trashed	Max length (nt)	Median length (nt)
GE	29,038	28,125	27,792	4%	830	631

HE	37,360	36,349	36,207	3%	1618	624

TE	40,412	39,700	39,236	3%	950	556

CE	20,514	20,209	20,131	2%	835	560

ME	28,856	28,131	27,859	3%	1,021	627

EN	22,383	22,128	22,079	1%	813	576

GC	21,245	20,976	20,897	2%	828	588

TC	20,916	20,529	20,468	2%	834	637

ES	12,193	11,791	10,804	11%	992	751

LE	15,125	14,468	12,091	20%	1,004	705

FL	6,498	5,735	5,160	21%	1,056	515

ST	12,181	11,783	11,324	7%	971	749

PS	7,557	7,231	7,224	4%	996	605

**Total**	**274,278**	**267,155**	**261,272**	**5%**	**1,618**	**613**

Minimum cut-off length for EST analysis was 80 nucleotides.

**Table 2:** Distribution of ESTs and unigenes (both contigs and singletons) in each library, and in the pooled data set (labeled Total)

Tissue library	Total ESTs in library	Number of clustered ESTs	Number of contigs	Number of singletons	Total number of unigenes per library	Number of contigs unique to library
GE	27,778	26,423	5,537	1,355	6,892	210

HE	36,197	34,151	6,148	2,046	8,194	298

TE	39,212	36,996	7,406	2,216	9,622	409

CE	20,121	19,122	4,501	999	5,500	164

ME	27,851	26,653	4,999	1,198	6,197	262

EN	22,074	21,093	4,504	981	5,485	175

GC	20,888	19,356	5,788	1,532	7,320	288

TC	20,453	19,174	5,371	1,279	6,650	289

ES	10,800	10,419	1,247	381	1,628	72

LE	12,085	11,419	1,860	666	2,526	145

ST	11,323	10,785	1,896	538	2,434	118

PS	7,224	6,112	3,287	1,112	4,399	275

FL	5,156	4,603	1,261	553	1,814	199

**Total**	**261,162**	**246,306**	**15,784**	**14,856**	**30,640**	

The last column states how many of the contigs were present in only one cDNA library, indicating potential tissue specific expression.

**Table 3:** GenBank accession numbers for the different flax EST libraries and their tissue source

GenBank Accession	Library Name	Tissue Source
LIBEST_026995	LUSGE1NG	Globular embryo

LIBEST_026996	LUSHE1NG	Heart embryo

LIBEST_026997	LUSHE1AD	Heart embryo

LIBEST_026998	LUSTE1NG	Torpedo embryo

LIBEST_026999	LUSTE1AD	Torpedo embryo

LIBEST_027000	LUSBE1NG	Cotyledon embryo

LIBEST_027001	LUSME1NG	Mature embryo

LIBEST_027002	LUSME1AD	Mature embryo

LIBEST_027003	LUSGC1NG	Globular seed coat

LIBEST_027004	LUSTC1NG	Torpedo seed coat

LIBEST_027005	LUSEN1NG	Endosperm pooled

LIBEST_027006	LUSFL1AD	Flower

LIBEST_027007	LUSES1AD	Etiolated seedling

LIBEST_027008	LUSLE1AD	Leaf

LIBEST_027009	LUSST1AD	Stem

LIBEST_027010	LUSPS1AD	Stem peel

LIBEST_027011	LUSST1MD	Stem

### GO annotation and functional categorization

**Figure 3:**
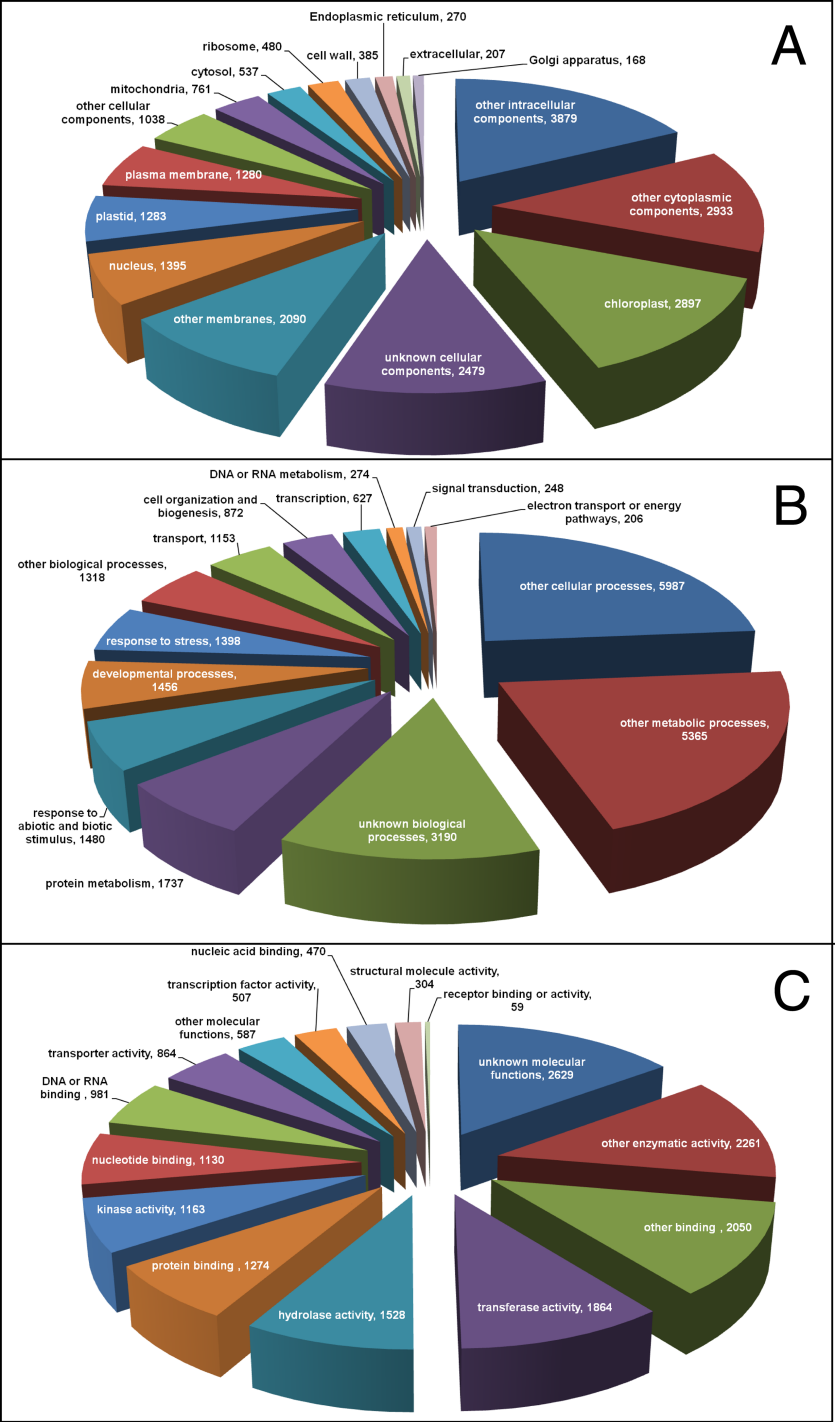
**GO annotation of flax unigenes**. TAIR annotation of flax unigenes indicates broad representation within each category. (A) Biological processes; (B) Molecular functions; (C) Cellular components. Numbers shown signify ESTs for each sub-category.

The unigene collection of 30,640 contigs and singletons was analyzed using the BLASTX algorithm against the UniProt-plants and TAIR databases. The unigenes that showed significant homology to known genes (E-value ≤ e-10) against UniProt-plants were selected for Gene Ontology (GO) annotation and further mapping of the GO terms to TAIR database which is manually and computationally curated on a ongoing basis [21]. The values generated for the different GO-categories were used to generate the classification based on molecular functions, biological processes and cellular components (Figure 3). Based on the BLAST analysis in TAIR, 23,418 unigenes showed significant homology to Arabidopsis genes and these are listed in a spreadsheet (Additional File 1; http://bioinfo.pbi.nrc.ca/portal/flax/) along with the distribution of ESTs for each unigene from the 13 tissue libraries. Our analysis suggests that the different GO-categories are well represented in our unigene dataset indicative of a broad coverage of expressed genes in the flax genome.

### Hierarchical cluster analysis of flax tissue based EST collections

**Figure 4:**
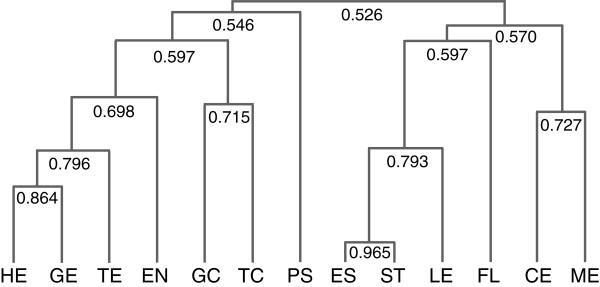
**Hierarchical cluster analysis of flax EST libraries**. Three gene expression clusters were identified, viz., early differentiating seed tissues, maturing embryos and juvenile vegetative tissues. The tree shows hierarchical clustering of the tissue-based libraries based on similarity/distance as measured by the Pearson correlation coefficient. Values close to 1 have high degree of similarity whereas lower values indicate the degree of distance between two libraries. Globular embryo (GE), heart embryo (HE), torpedo embryo (TE), cotyledon embryo (CE), mature embryo (ME), globular stage seed coat (GC), torpedo stage seed coat (TC), pooled endosperm (EN), etiolated seedlings (ES), stem (ST), stem peel (PS), leaves (LF), and mature flower (FL).

In order to compare the gene expression profile in different tissues, the entire set of 261,272 EST sequences was subjected to hierarchical cluster analysis using the software HCE3.5 [22] (see Methods). Amongst the parameters required for hierarchical cluster analysis, we selected the average linkage method and the Pearson correlation coefficient for the similarity/distance measure, a technique which has been widely used in microarray analysis [23]. The results are shown in Figure 4. The analysis shows that in general gene expression is most closely related in tissues that are developmentally related and connected. For example, globular (GE) and heart (HE) embryo stages are most closely related, followed closely by the torpedo stage (TE). The maturing embryos, viz., cotyledon (CE) and mature (ME) stages clustered together but were distantly placed from the early stage embryos. The two seed coat stages (GC and TC) also shared a relatively high degree of similarity to each other. Gene expression in the pooled endosperm tissue (EN) from early developing seed stages shared some similarity with early embryonic stages but was more distant from the seed coats and maturing embryos. It is interesting to note that the CE and ME stages cluster away from the early seed tissues (GE, HE, TE, GC, TC and EN) and to a lesser extent from other non-seed tissues viz., (ES, LE, FL, ST) which is indicative of the distinct seed maturation program that is occurring in the later stages of embryo development. As the stem peel (PS) did not contain all of the tissues normally present in whole stems (ST), and was enriched for the phloem and phloem fiber cells [24], the PS gene expression profile did not cluster with ST, and as expected was distantly placed from the rest of the vegetative tissues and seed tissues. Whole stems (ST) and etiolated seedlings (ES) showed a high degree of similarity, possibly due to their polysaccharide composition. Both whole stems and etiolated seedlings are likely to be particularly enriched in xylem tissues, the secondary walls of which produce polysaccharides different from those found in the pectin-enriched phloem fibers in (PS), seed coats (GC, TC), or the primary walls of developing embryos [25]. Taken together, this analysis showed three distinct patterns of relatedness of gene expression among the 13 tissues: early seed stages, the maturing embryo stages and the juvenile vegetative tissues (ES, ST and LF).

### Nearly a fifth of the identified transcriptome is apparently unique to flax

To identify the degree of potential homology of the flax unigenes shared with other plant species, we performed BLASTX analysis against the proteomes representing the six fully sequenced and annotated genomes of Arabidopsis, *Oryza sativa *(rice), *Sorghum bicolor *(sorghum), *Vitis vinifera *(grape), *Populus trichocarpa *(poplar) and *Ricinus communis *(castor bean) (see Methods). In general, the deduced flax polypeptides are more similar to those of poplar and castor bean than to grape, Arabidopsis, sorghum or rice (Table 4). This is consistent with the taxonomic grouping of flax, poplar and castor bean within the order Malpighiales [26]. The order Malpighiales, which is a large diverse grouping of 42 families containing several economically important species, is hypothesized to have diverged within a relatively short time frame and the taxonomic relationship of families within this order is poorly resolved. However, genome sequencing of poplar [27], castor bean [28], cassava [29] and large EST libraries from other species within this order including flax (this study) will likely aid in molecular systematic studies to address broader phylogenetic relationships between these families. Whereas 66% of the unigenes (20,251) had hits in all six species, 16.8% (5,152) of the unigenes had no hits in any species, indicating that they may be flax specific genes.

**Table 4:** Flax unigenes are most similar to poplar and castor bean genes

	Confidence level			
**Species**	**x ≥ e**^**-19**^ **(low)**	**e**^**-20 **^**≥ × ≥ e**^**-49**^ **(medium)**	**e**^**-50 **^**≥ × ≥ e**^**-98**^ **(high)**	**x ≤ e**^**-99**^ **(highest)**

Poplar	3,638	8,740	10,002	2,308

Castor Bean	4,051	8,407	9,926	2,274

Grape	3,844	8,773	9,517	2,013

Arabidopsis	4,140	8,958	9,039	1,881

Sorghum	4,586	9,056	7,828	1,465

Rice	4,514	9,046	7,892	1,459

Number of blast hits (BLASTX) of the 30,640 flax unigenes against six different plant genomes. Blast hit blocks indicate the confidence level with which the flax unigenes match other species' genes.

### Key embryogenesis regulators are present in the EST collections

**Figure 5:**
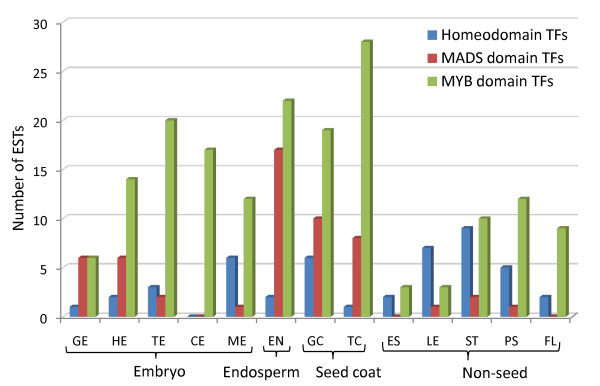
**Distribution of putative flax unigenes encoding MADS, homeodomain and MYB domain transcription factors**. These transcription factor families are expected to have wide distribution and are found in majority of the flax EST libraries. EST distribution of flax unigenes used to compile this graph is listed in Additional File 2.

Transcription factors (TFs) are generally expressed at low levels and their presence in ESTs indicate the depth of the EST coverage. We analyzed the TFs present in all flax libraries. Among the TF families, three important motifs present in the TFs that regulate plant growth and development are the homeodomain (HD), MADS and the MYB domain [30]. TFs containing these domains are well represented in the 13 libraries and indicate good coverage of low expressed genes in the EST datasets (see Figure 5; Additional File 2). Overall, at least 783 transcription factors are present in the 30,640 flax unigenes.

**Figure 6:**
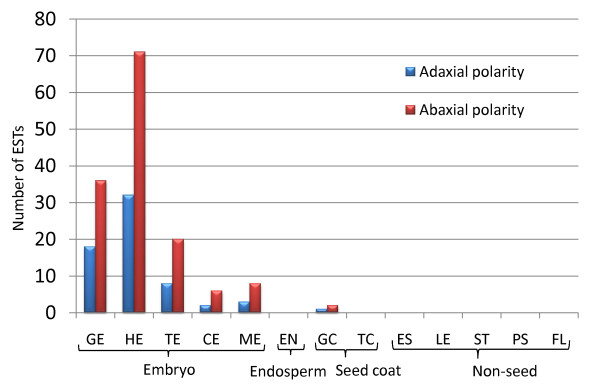
**Putative flax unigenes representing organ polarity transcription factors**. Organ polarity transcription factor ESTs are most abundant during cotyledon primordia differentiation of heart-stage embryos. Adaxial (*HD-ZIPIII *family and *AS1*) and abaxial (*YABBY *and *KANADI *families) gene expression establishes organ polarity. EST distribution of flax unigenes used to compile this graph is listed in Additional File 2.

As one of the main objectives of this study was to gain a better understanding of what happens in the flax seed as it develops, we further analyzed the EST libraries for transcription factors with specific roles in embryo and seed development (Additional File 2). The establishment of the adaxial and abaxial polarity during cotyledon primordia differentiation at the heart stage of embryo development is specified by the HD-ZIPIII family, *ASYMMETRIC LEAVES1 *(*AS1*) (adaxial) and *YABBY, KANADI *families (abaxial) respectively [31]. ESTs corresponding to adaxial and abaxial polarity specifying TFs are expressed from globular stage onwards with maximum number of ESTs in the heart stage when the cotyledon primordia are specified (Figure 6; Additional File 2).

**Figure 7:**
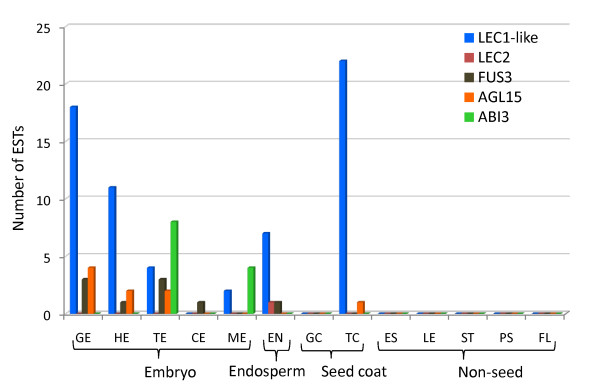
**Putative flax unigenes encoding transcription factors that are known embryogenesis regulators**. Tissue distribution of flax unigenes encoding ESTs with similarity to important regulators of embryogenesis are present in developing flax seed tissue libraries, and not in non-seed libraries. EST distribution of flax unigenes used to compile this graph is listed in Additional File 2.

*LEAFY COTYLEDON *(*LEC*) genes *LEC1, LEC1-like *(*L1L*), *LEC2 *and *FUSCA3 *(*FUS3*) are master regulators of embryogenesis that are primarily expressed throughout seed development, and ectopic expression of these TFs results in somatic embryogenesis or embryonic characteristics being overlaid on vegetative organs [32-35]. *ABI3 *is expressed only during seed maturation and is a key regulator of seed maturation processes such as seed dormancy and storage reserve accumulation [36]. *AGAMOUS-LIKE15 *(*AGL15*), a MADS domain containing TF is primarily expressed during Arabidopsis seed development and its ectopic expression increases the competency of cells to respond to somatic embryogenesis induction conditions [37,38]. In Arabidopsis, *AGL15 *is directly upregulated by *LEC2 *[39]. In addition, *LEC2, FUS3 *and *ABI3 *have all been demonstrated to be direct targets of AGL15 [40]. Examination of flax unigenes showed seed-specific enriched expression of *L1L, LEC2, FUS3, ABI3 *and *AGL15 *(Figure 7; Additional File 2). Only one EST with similarity to *LEC2 *was identified. The absence of *LEC1 *and the presence of the closely related *L1L *in seed tissues have also been observed for scarlett runner bean [33]. The identification of ESTs in seed-specific libraries that are pertinent to seed maturation program lends support to the quality of these libraries.

### Mining for biochemical pathway-specific ESTs that make flax seed nutritionally rich

The flax seed contains many nutritionally important compounds such as proteins, fatty acids, lignans, flavonoids and mucilage. To determine the usefulness of the EST resources generated in this study, we queried for genes involved in the synthesis of the above noted seed components. In order to identify potential candidate enzymes amongst many flax unigenes, the Additional Files 3 and 4 provide the first step to narrow down putative flax candidates by examining the timing and distribution of ESTs across different tissues.

#### Seed storage proteins

**Figure 8:**
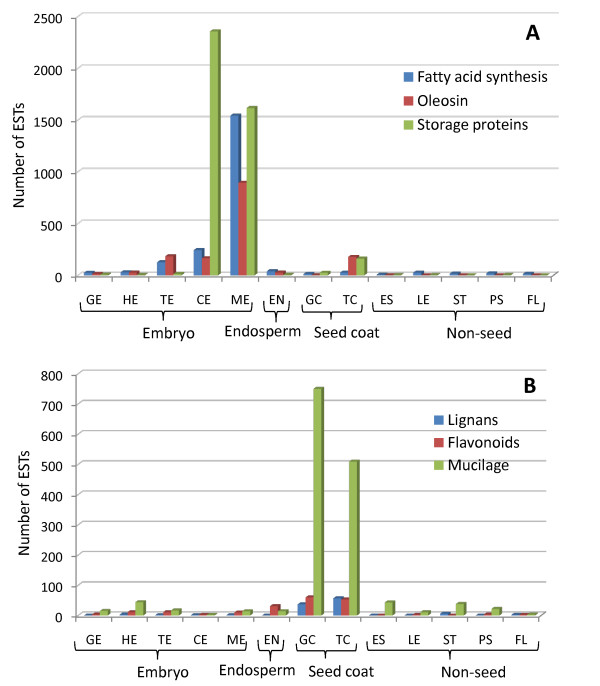
**EST distribution across tissue libraries of biosynthetic genes of important flax seed nutritional components**. Fatty acid biosynthesis, oleosin oil body proteins and storage protein ESTs are highly represented in zygotic library compartments (A). Lignan, flavonoid and mucilage biosynthetic pathways are highly represented in maternal seed coat compartments (B). EST distribution of flax unigenes used to compile these graphs is listed in Additional File 3 and Additional File 4.

Much of the proteins in flax seeds are storage proteins that exist within protein storage vacuoles and these proteins constitute 23% of the whole flax seed [41]. Storage proteins in flax seed are made up of ~65% globulins and ~35% albumins [11]. Conlinin is a 2S albumin and cupin and cruciferin are 11S and 12S globulins, respectively. Our EST data correlates the expression of the genes coding for the storage proteins with the reported levels of proteins in flax seeds (Figure 8A; Additional File 3). Globulin encoding genes were expressed at much higher levels than those encoding the albumin and were observed in the later cotyledon (CE) and mature (ME) stages of embryo development. Interestingly, small numbers of ESTs for all the storage proteins were identified in young seed coats, primarily at the torpedo stage (Figure 8A; Additional File 3). This is in agreement with the observation that a conlinin gene promoter is active in early stages of seed coat development [42]. Pooled endosperm from the corresponding seed coat stages did not identify any storage protein ESTs. These observations suggest that the seed coat does have a role in storage protein synthesis. Given that the seed coat is a major part of the overall mass in developing seeds, the seed coat might be a transient source of protein for developing embryos.

#### Fatty acids and oil body formation

**Figure 9:**
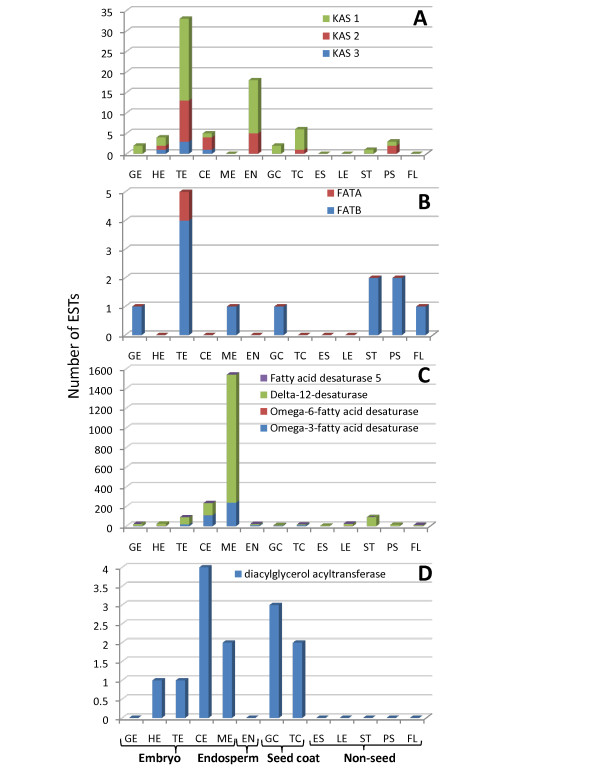
**EST distribution of fatty acid biosynthetic genes during seed development and maturation across tissue libraries**. (A) acyl chain elongation (Keto Acyl Synthases); (B) acyl chain termination (Fatty Acyl Thioesterases); (C) desaturation (Desaturases); (D) triacylglycerol (TAG) biosynthesis. EST distribution of flax unigenes used to compile these graphs is listed in Additional File 3.

Mature flax seeds consist of approximately 43% oil, mostly in the form of triacylglycerols (TAGs) within oil bodies located in the embryo [11]. In order to study the timing and source of lipid synthesis within the developing seeds, enzymes representing the four key steps of fatty acid synthesis were studied: acyl-chain elongation, termination, desaturation and TAG synthesis [43,44] (Figure 8A, Figure 9; Additional File 3). Based on the preponderance of ESTs representing the 3-ketoacyl-acyl carrier protein synthases (KAS1, KAS2 and KAS3) in the various tissues, it appears that acyl chain elongation activity increases during the torpedo stage and that the embryo, endosperm and seed coat all contribute to this activity in the seed (Figure 9A). Although the number of ESTs representing termination of elongation by fatty acyl-ACP thioesterases (FATA and FATB) was lower than KAS ESTs, this activity also appears to peak during the torpedo stage (Figure 9B). Within the developing embryos, fatty acids are transferred onto a glycerol backbone to form triacylglycerols by the activity of diacylglycerol acyltransferase (DGAT). TAGs are stored in oil bodies, the outer membrane of which is a spherical phospholipid monolayer interspersed with the protein oleosin [44]. ESTs representing DGAT were found in quantities similar to the *FATA *and *FATB *ESTs, i.e. in very low quantities. The key difference is that this activity seems to peak later, during the cotyledon embryonic stage rather than the torpedo stage (Figure 9D). Also, while termination of elongation and release of free FAs appears to occur in both seed tissues as well as in some of the vegetative tissues, DGAT expression in vegetative tissues is too low to detect with the EST counts. Desaturation is the key step that results in the desirable omega-3 and omega-6 fatty acids [44]. This seems to occur later during seed development as the spike in the number of ESTs representing the *Fatty Acid Desaturases *(*FAD) 2, 3, 5 *and *8 *occurs within the mature embryo (Figure 9C). One of the omega-3 fatty acids found in flax, alpha-linolenic acid (ALA, 18:3n-3), constitutes up to 55% of the total seed oil [41]. ALA is an essential fatty acid in human diet and it is converted to eicosapentaenoic acid (EPA) and docosahexaenoic acid (DHA) which are then incorporated into membrane phospholipids. Some fatty acids are used in plant membrane synthesis, wax formation and pigmentation. The repertoire of lipid synthesis ESTs found in stem, stem peel and flowers provide a basis to probe these processes in these tissues (Figures 8A and 9).

Oleosins, proteins associated with oilbodies, are known to stabilize them by preventing the coalescence of the lipid particles during seed germination [45]. In our datasets, the expression of putative homologs of Arabidopsis *Oleosin 1, 2 *and *3 *genes was observed in the embryo beginning at the torpedo stage (TE), with greater levels in mature stage (ME) (Figure 8A; Additional File 3). This also coincides with the expression in the CE and ME stages of the *FAD *desaturases that are involved in the formation of the omega-3 and omega-6 fatty acids. *Oleosin *gene expression has been shown to be regulated in part by ABI3 in Arabidopsis [46]. There is also a correlation of *ABI3 *with *oleosin *ESTs at the torpedo and mature embryo stages (Figure 7 and 8A; Additional File 2 and 3), indicating that the EST data is reflective of the underlying genetic and biochemical programs.

#### Lignans

Flax is a rich source of secoisolariciresinol diglycoside (SDG). SDG is converted by intestinal bacteria to the so-called mammalian lignans enterodiol and enterolactone. SDG has phytoestrogen, antioxidant, and anticancer activities [8]. Lignans present in the seed coat of flax and are derived from coniferyl alcohol by the initial action of oxidases and dirigent proteins that yield pinoresinol [47]. Sequential reduction of pinoresinol by pinoresinol-lariciresinol reductase (PLR) results in the formation of SDG [48]. Analysis of our flax unigene collection identified several candidates corresponding to dirigent proteins and PLR that are predominantly expressed in the globular and torpedo stage seed coats (Figure 8B; Additional File 4). Dirigent proteins had a higher number of EST hits in globular stage seed coat which corresponds with its early role in the lignan biosynthetic pathway, whereas pinoresinol-lariciresinol reductase, which acts later in the pathway, is expressed in the seed coat at the torpedo stage.

#### Flavonoids

Flavonoids constitute a major class of plant phenolics. Flax seeds are a rich source of flavonoids, which includes flavonols and anthocyanidins [49]. The flavonoid biosynthesis branch starts with the formation of chalcone, a reaction catalyzed by chalcone synthase (CHS), followed by the synthesis of flavanone by chalcone isomerase (CHI). Dihydroflavonol reductase (DFR) activity is the committing step for leucoanthocyanidin synthesis and proanthocyanidin, anthocyanidin and anthocyanin synthesis follows this step [50]. The key enzymes in the flavonoid synthesis pathway, viz., CHS, CHI and DFR are expressed during flax seed development especially in the seed coat tissues as shown by the number of ESTs (Figure 8B; Additional File 4). *BANYULS *(*BAN*) gene of Arabidopsis encodes an anthocyanidin reductase in the anthocyanidin branch that produces cis-3-flavan-3-ol which has known health benefits in humans [51]. ESTs representing *BAN *are present in the embryonic and seed coat tissues of flax indicating that flax seeds could be a likely source of cis-3-flavan-3-ols (Figure 8B; Additional File 4).

#### Mucilage synthesis and secretion

During flax seed development, the ovule integuments differentiate and form specialized cell types which include the seed coat epidermis that stores mucilaginous compounds. The chemical composition of flax seed mucilage has been investigated because of its benefits to human health. The pectin rhamnogalacturonan I (RG I) is the primary constituent of seed mucilage in Arabidopsis and several other species, whereas flax seed mucilage contains a mixture of neutral arabinoxylans (75%) and RG I (25%) [52-54]. In the mature seed, the cells of the outer epidermal layer of the seed coat are transformed into mucilage secretory cells (MSCs) that release mucilage upon seed hydration. In Arabidopsis, *MUCILAGE-MODIFIED4 *(*MUM4*) gene encodes Rhamnose Synthase2, an enzyme that catalyzes the synthesis of RG I [55], whereas *MUM2 *encodes a beta-galactosidase that enables the hydration properties of the mucilage by modifying the RG I side chains [56]. Furthermore, *AtBXL1 *gene, which encodes a beta-xylosidase/alpha-arabinofuranosidase, is essential for the release of mucilage by degradation of the arabinan side chains in the mucilage and/or cell wall of the mucilage secretory cells [57]. Genes encoding rhamnose synthase and beta-xylosidase are represented in the GC and TC tissue specific ESTs indicating that the mucilage synthesis and secretion pathway observed in Arabidopsis is represented in flax and the expression of corresponding genes are enriched specifically in seed coat tissues (Figure 8B; Additional File 4). However, ESTs corresponding to the rhamnose synthase did not include the ortholog of Arabidopsis *MUM4 *gene, suggesting the possibility that there is some diversity of this mucilage synthesis pathway in flax. Galacturonosyltransferases that are involved in the polymerization of galacturonic acid [58] to form pectic RG I were also well represented in GC and TC tissue specific manner, indicative of their conserved roles in the synthesis of mucilage in the seed coat (Figure 8B; Additional File 4). Interestingly, ESTs corresponding to the putative homologs of the *AtBXL2 *gene, a member of the small gene family that includes *AtBXL1 *[57], were expressed at very high levels in the seed coat tissues suggesting their role in the quick and uniform release of mucilage from the flax seed coat upon imbibition (Figure 8B; Additional File 4). A putative flax ortholog of *AtBXL1 *is also one of the most abundant ESTs identified in a previous report of cDNAs from fiber-bearing flax tissues [59].

## Conclusions

We have developed a comprehensive EST resource for flax representing developmental stages of specific seed tissues, some vegetative and reproductive tissues. These resources include publicly available EST sequences at GenBank (Table 3), a queryable flax unigene database (http://bioinfo.pbi.nrc.ca/portal/flax/) and unigene distribution across libraries (Additional File 1). The datasets developed in this study enhance the genomic resource base for flax, an important crop. These resources can contribute to gene discovery and development of expanded molecular marker sets for breeding. Additionally, the unigene set developed in this study will contribute to the annotation and assembly of the whole flax genome sequence.

The recently published flax-specific microarray based on EST sequences obtained from a fiber focused study while the present manuscript was under preparation provides a complimentary genomic tool for flax gene expression analysis [60]. However, having the EST resources of the developing seed partitioned into embryo, endosperm, and seed coat compartments relative to vegetative tissues in our study allows further refinement into determining the involvement of genes in temporally and spatially specific metabolic pathways. Analysis of our datasets indicates good representation of biological processes related to seed development. 7,222 flax unigenes did not have homologs to the genes of the model species Arabidopsis and there were 5,152 unigenes that do not show any homology to plant species in UniProt. These 5,152 unigenes therefore likely represent flax-specific genes. Many of these unidentified genes were broadly distributed whereas some were specific to a single tissue. Further studies of these will provide new insights into flax-specific programs.

## Materials and methods

### Plant growth conditions and tissue collection

Breeder seed (F11) of *Linum usitatissimum *cv CDC Bethune was selfed for 7 generations (F18) as single plants in the Phytotron at the University of Saskatchewan. F19 seeds were germinated and grown in a growth chamber using a daily cycle consisting of 16 hours of light (23°C) and 8 hours of dark (16°C). Tissue samples were collected and frozen immediately in liquid nitrogen. The leaf, stem and flower samples were each collected from more than 10 individual plants. Dissection of 5,000 flax seeds was performed in order to isolate sufficient endosperm, embryonic, and seed coat tissues for creating the cDNA libraries. Five stages of embryos representing globular, heart, torpedo, cotyledon, and mature stages were isolated from developing seeds. Seed coat samples were collected from globular and torpedo embryo stages. Endosperm tissues were pooled from seeds containing globular to torpedo embryo stages. Etiolated seedlings were generated by incubating seeds on MS medium plates in the dark for four days and prior to harvesting, the seed coats were removed. The stem peel tissue consisting of epidermis, cortical tissues, phloem, developing fibers, and cambial tissue was prepared from stems of four week-old *Linum usitatissimum *L. cv Norlin germinated and grown as described previously [24].

### RNA isolation and cDNA library construction

The stem peel library (PS) was constructed using the Superscript Plasmid System with Gateway Technology for cDNA Synthesis and Cloning (Invitrogen, Carlsbad, CA) [24]. cDNAs were directionally cloned in pCMV-SPORT6 (Invitrogen) and transformed in chemically competent DH5α-FT *E. coli*. For the remaining 12 libraries, total RNA was isolated using the RNeasy Plant Mini Kit (Qiagen, Cat. No. 74904). On-column DNase digestion was performed using the RNase-free DNase set (Qiagen, Cat. No. 79254). Approximately 2 μg of total RNA from the tissues was used to construct each cDNA library. These 12 libraries were constructed using the Creator SMART cDNA library construction kit (Clontech, Cat. N. 634903). The 8 libraries derived from seed tissues (globular, heart, torpedo, cotyledon and mature embryos, as well as endosperm and globular and torpedo stage seed coats) were prepared as per the manual instructions and are in the pDNR-lib vector (Clontech).

Two modifications to the manual were made during construction of the cDNA libraries for leaf, stem, flower and etiolated seedling. First, the cDNA size fractionation was performed on agarose gel instead of CHROMA SPIN-400 column supplied by the kit. The SfiI digested cDNAs were loaded into a 1% TAE agarose gel, and run for about 2 cm. The cDNA samples were excised from the agarose gel and purified using the QIAquick Gel Extraction kit (Qiagen, Cat. No. 28704). Second, a modified pBluescript II SK(+) vector was used. A *ccdb *gene, with SfiI sites at both ends, was inserted between the EcoRI and XhoI of pBluescript II SK(+). This modified vector was then digested with SfiI, agarose gel purified, and used for ligation with the SfiI digested cDNA samples. Ligations to construct the libraries were performed according to the Creator SMART manual.

### EST sequencing and analysis

The libraries were spread onto the LB medium plates and cultured at 37°C overnight. Individual clones were picked into 96 or 384-plates manually or automatically by a Colony Picker (CP-7200, Norgren Systems). The ESTs were sequenced on the ABI 3730xl DNA Analyzer (Applied Biosystems) at the DNA sequencing facility of the National Research Council-Plant Biotechnology Institute (NRC-PBI, Saskatoon, SK, Canada). The HE, TE and ME libraries were sequenced in two batches (Table 3). A total of 274,278 sequences were obtained. The reader can refer to Table 1 for the tissue distribution. The assembling process of EGassembler was used. Details are given in the EGassembler tutorial [18] (http://egassembler.hgc.jp/cgi-bin/eassembler4.cgi?pmode=help&i_param=tutorial). In the first step, the sequences were cleaned and ones with length of less than 100 bases were removed. The following steps consisted of masking the repeats, vector and organelle sequences. Masked nucleotides were removed and any resulting sequences less than 80 bases in length were also removed. The first clustering process was performed for each separate library. The resulting 78,209 sequences (27,168 contigs and 51,041 singletons) were then merged, and reassembled, resulting in 30,640 unigenes (15,784 contigs and 14,856 singletons). These unigenes were reallocated back into their respective individual libraries. All EST sequences and unigenes have been deposited at http://bioinfo.pbi.nrc.ca/portal/flax/. The clustering of the ESTs were performed using Hierarchical Clustering Explorer 3.5 sofware (http://www.cs.umd.edu/hcil/hce/power/power.html) [22]. The number of EST reads for each unigene in each of the 13 different tissues was used as the input data for HCE3.5 software with parameters set for Pearson correlation coefficient for similarity/distance measure and average linkage method for hierarchical clustering.

BLASTX analysis of flax unigenes against the six plant genomes were performed using the proteomes from the respective species: *Arabidopsis thaliana *'ftp://ftp.arabidopsis.org/home/tair/Genes/TAIR9_genome_release/TAIR9_sequences/'; *Oryza sativa *'ftp://ftp.plantbiology.msu.edu/pub/data/Eukaryotic_Projects/o_sativa/annotation_dbs/pseudomolecules/version_6.1/all.dir/'; *Populus trichocarpa *'http://genomeportal.jgi-psf.org/Poptr1_1/Poptr1_1.download.ftp.html'; *Vitis vinifera *'http://www.uniprot.org/uniprot/?query=taxonomy:29760&format=*'; *Sorghum bicolor *'ftp://ftp.jgi-psf.org/pub/JGI_data/phytozome/v5.0/Sbicolor/annotation/Sbi1.4/Sbi1.4.pep.fa.gz'; and *Ricinus communis *from swissprot.

### Microscopy

#### Clearing

Fertilized ovules were cleared for 2 days in chloral hydrate solution (8:1:2 chloral hydrate - glycerol - water w/v/v) and viewed with a compound microscope (Leica DMR) using Nomarski optics.

#### Scanning Electron Microscopy

Samples were fixed in 3% glutaraldehyde, post-fixed in 1% osmium tetroxide and dehydrated in a graded acetone series as described [61]. Samples were mounted on aluminum stubs and coated with gold in an Edwards S150B sputter coater. Observations were made with a Phillips 505 scanning electron microscope at 30 kV and recorded on Fujifilm FP-100b professional film. Images were scanned and treated in Adobe Photoshop CS (Adobe Systems, San Jose, California) to improve the contrast and place scale bars.

## Authors' contributions

PV, DQ, SQ and RD: conception, design, experiments, data analysis, interpretation and writing of manuscript; SLS and MAM: analysis, interpretation and writing of manuscript; CT, DC, JN and EW: bioinformatic analysis of datasets; MD: stem peel cDNA library and analysis; FB, AS and SC: coordination and interpretation; GR and GS: interpretation, important intellectual contribution and writing of manuscript. All authors read, commented and approved the manuscript.

## Supplementary Material

Additional File 1 **Number of ESTs representing flax unigenes distributed across 13 libraries**. Annotation of these unigenes was based on the Arabidopsis genome. Click here for file

Additional File 2 **Number of ESTs associated with the transcription factors distributed across 13 libraries**. Click here for file

Additional File 3 **Number of flax ESTs associated with seed storage reserve pathways distributed across 13 libraries**. The unigenes were selected based on Arabidopsis gene annotations, except for conlinin which was based on UniProt database. Click here for file

Additional File 4 Number of ESTs associated with flax lignan, flavonoid and mucilage pathways distributed across 13 libraries Click here for file

## References

[B1] Vaisey-Genser M Morris DH Muir A, Westscott N History of cultivation and uses of flaxseed Flax, The genus Linum 2001 Amsterdam: Hardwood Academic Publishers 1 21

[B2] Diederichsen A Richards K A. M, Westscott N Cultivated flax and the genus Linum L.: Taxonomy and germplasm conservation Flax, The genus Linum 2001 Amsterdam: Hardwood Academic Publishers 22 54

[B3] Bennett MD Leitch IJ Plant DNA C-values database (release 3.0) 2004 http://www.rbgkew.org.uk/cval/homepage.html

[B4] Cullis CA DNA sequence organisation in the flax genome Biochimica et Biophysica Acta (BBA) - Nucleic Acids and Protein Synthesis 1981 652 1 15 10.1016/0005-2787(81)90203-3 7213728

[B5] Daun JK DeClercq DR Sixty years of Canadian flaxseed quality surveus at the Grain Research Laboratory Proc of the Flax Institute of the United States 1994 Fargo, ND.: Flax Institute of the United States 192 200

[B6] Thompson LU Rickard SE Cheung F Kenaschuk EO Obermeyer WR Variability in anticancer lignan levels in flaxseed Nutrition and Cancer 1997 27 26 30 10.1080/01635589709514497 8970178

[B7] Westcott ND Muir AD Variation in the concentration of the flaxseed lignan concentration with variety, location and year Proc of the Flax Institute of the United States 1996 56 Fargo, ND: Flax Institute of the United States 77 80

[B8] Touré A Xueming X Flaxseed Lignans: Source, Biosynthesis, Metabolism, Antioxidant Activity, Bio-Active Components, and Health Benefits Comprehensive Reviews in Food Science and Food Safety 2010 9 261 269 10.1111/j.1541-4337.2009.00105.x 33467817

[B9] Vaisey-Genser M Morris DH Flaxseed: Health, Nutrition and Functionality 1997 Winnipeg, MB.: Flax Council of Canada

[B10] Oomah BD Mazza G Flaxseed proteins--a review Food Chemistry 1993 48 109 114 10.1016/0308-8146(93)90043-F

[B11] Westcott ND Muir AD Chemical studies on the constituents of Linum spp Flax, the Genus Linum 2001 Amsterdam: Hardwood Academic Publishers

[B12] Chung MWY Lei B Li-Chan ECY Isolation and structural characterization of the major protein fraction from NorMan flaxseed (Linum usitatissimum L.) Food Chemistry 2005 90 271 279 10.1016/j.foodchem.2003.07.038

[B13] Cloutier S Niu Z Datla R Duguid S Development and analysis of EST-SSRs for flax (Linum usitatissimum L.) Theor Appl Genet 2009 119 53 63 10.1007/s00122-009-1016-3 19357828

[B14] Cullis CA Kolle C Flax Genome mapping and molecular breeding in plants - Oilseeds 2007 2 Berlin Heidelberg: Springer-Verlag

[B15] Capron A Chatfield S Provart N Berleth T Embryogenesis: Pattern Formation from a Single Cell The Arabidopsis Book 2008 The American Society of Plant Biologists 1 28 10.1199/tab.0126 PMC3243344 22303250

[B16] Ellis PR Kendall CW Ren Y Parker C Pacy JF Waldron KW Jenkins DJ Role of cell walls in the bioaccessibility of lipids in almond seeds The American Journal of Clinical Nutrition 2004 80 604 613 15321799 10.1093/ajcn/80.3.604

[B17] Sachs J Vorlesungen uber pflanzen-physiologie, Verlag Wilhem Engelmann, Leipzig 1887 http://www.seedbiology.de/structure.asp#ricinus

[B18] Masoudi-Nejad A Tonomura K Kawashima S Moriya Y Suzuki M Itoh M Kanehisa M Endo T Goto S EGassembler: online bioinformatics service for large-scale processing, clustering and assembling ESTs and genomic DNA fragments Nucleic Acids Research 2006 34 W459 W462 10.1093/nar/gkl066 16845049 PMC1538775

[B19] Bennett MD Leitch IJ Price HJ Johnston JS Comparisons with Caenorhabditis (~100 Mb) and Drosophila (~175 Mb) Using Flow Cytometry Show Genome Size in Arabidopsis to be ~157 Mb and thus ~25% Larger than the Arabidopsis Genome Initiative Estimate of ~125 Mb Annals of Botany 2003 91 547 557 10.1093/aob/mcg057 12646499 PMC4242247

[B20] TAIR 2009 http://www.arabidopsis.org/portals/genAnnotation/gene_structural_annotation/annotation_data.jsp

[B21] Berardini TZ Mundodi S Reiser L Huala E Garcia-Hernandez M Zhang P Mueller LA Yoon J Doyle A Lander G Functional Annotation of the Arabidopsis Genome Using Controlled Vocabularies Plant Physiol 2004 135 745 755 10.1104/pp.104.040071 15173566 PMC514112

[B22] Seo J Gordish-Dressman H Hoffman EP An interactive power analysis tool for microarray hypothesis testing and generation Bioinformatics 2006 22 808 814 10.1093/bioinformatics/btk052 16418236

[B23] Thalamuthu A Mukhopadhyay I Zheng X Tseng GC Evaluation and comparison of gene clustering methods in microarray analysis Bioinformatics 2006 22 2405 2412 10.1093/bioinformatics/btl406 16882653

[B24] Roach MJ Deyholos MK Microarray analysis of flax (Linum usitatissimum L.) stems identifies transcripts enriched in fibre-bearing phloem tissues Mol Genet Genomics 2007 278 149 165 10.1007/s00438-007-0241-1 17503083

[B25] Gorshkova TA Wyatt SE Salnikov VV Gibeaut DM Ibragimov MR Lozovaya VV Carpita NC Cell-Wall Polysaccharides of Developing Flax Plants Plant Physiol 1996 110 721 729 12226214 10.1104/pp.110.3.721 PMC157770

[B26] Wurdack KJ Davis CC Malpighiales phylogenetics: Gaining ground on one of the most recalcitrant clades in the angiosperm tree of life Am J Bot 2009 96 1551 1570 10.3732/ajb.0800207 21628300

[B27] Tuskan GA DiFazio S Jansson S Bohlmann J Grigoriev I Hellsten U Putnam N Ralph S Rombauts S Salamov A The Genome of Black Cottonwood, Populus trichocarpa (Torr. & Gray) Science 2006 313 1596 1604 10.1126/science.1128691 16973872

[B28] Chan AP Crabtree J Zhao Q Lorenzi H Orvis J Puiu D Melake-Berhan A Jones KM Redman J Chen G Draft genome sequence of the oilseed species Ricinus communis Nat Biotech 2010 28 951 956 10.1038/nbt.1674 PMC2945230 20729833

[B29] Cassava Genome Project 2010 http://www.phytozome.net/cassava

[B30] Riechmann JL Ratcliffe OJ A genomic perspective on plant transcription factors Current Opinion in Plant Biology 2000 3 423 434 10.1016/S1369-5266(00)00107-2 11019812

[B31] Bowman JL Eshed Y Baum SF Establishment of polarity in angiosperm lateral organs Trends in Genetics 2002 18 134 141 10.1016/S0168-9525(01)02601-4 11858837

[B32] Lotan T Ohto Ma Yee KM West MAL Lo R Kwong RW Yamagishi K Fischer RL Goldberg RB Harada JJ Arabidopsis LEAFY COTYLEDON1 Is Sufficient to Induce Embryo Development in Vegetative Cells Cell 1998 93 1195 1205 10.1016/S0092-8674(00)81463-4 9657152

[B33] Kwong RW Bui AQ Lee H Kwong LW Fischer RL Goldberg RB Harada JJ LEAFY COTYLEDON1-LIKE Defines a Class of Regulators Essential for Embryo Development Plant Cell 2003 15 5 18 10.1105/tpc.006973 12509518 PMC143447

[B34] Stone SL Kwong LW Yee KM Pelletier J Lepiniec L Fischer RL Goldberg RB Harada JJ LEAFY COTYLEDON2 encodes a B3 domain transcription factor that induces embryo development Proceedings of the National Academy of Sciences of the United States of America 2001 98 11806 11811 10.1073/pnas.201413498 11573014 PMC58812

[B35] Gazzarrini S Tsuchiya Y Lumba S Okamoto M McCourt P The Transcription Factor FUSCA3 Controls Developmental Timing in Arabidopsis through the Hormones Gibberellin and Abscisic Acid Developmental Cell 2004 7 373 385 10.1016/j.devcel.2004.06.017 15363412

[B36] Parcy F Valon C Raynal M Gaubier-Comella P Delseny M Giraudat J Regulation of Gene Expression Programs during Arabidopsis Seed Development: Roles of the ABI3 Locus and of Endogenous Abscisic Acid Plant Cell 1994 6 1567 1582 7827492 10.1105/tpc.6.11.1567 PMC160544

[B37] Heck GR Perry SE Nichols KW Fernandez DE AGL15, a MADS Domain Protein Expressed in Developing Embryos Plant Cell 1995 7 1271 1282 7549483 10.1105/tpc.7.8.1271 PMC160950

[B38] Harding EW Tang W Nichols KW Fernandez DE Perry SE Expression and Maintenance of Embryogenic Potential Is Enhanced through Constitutive Expression of AGAMOUS-Like 15 Plant Physiol 2003 133 653 663 10.1104/pp.103.023499 14512519 PMC219041

[B39] Braybrook SA Stone SL Park S Bui AQ Le BH Fischer RL Goldberg RB Harada JJ Genes directly regulated by LEAFY COTYLEDON2 provide insight into the control of embryo maturation and somatic embryogenesis Proceedings of the National Academy of Sciences of the United States of America 2006 103 3468 3473 10.1073/pnas.0511331103 16492731 PMC1413938

[B40] Zheng Y Ren N Wang H Stromberg AJ Perry SE Global Identification of Targets of the Arabidopsis MADS Domain Protein AGAMOUS-Like15 Plant Cell 2009 21 2563 2577 10.1105/tpc.109.068890 19767455 PMC2768919

[B41] DeClercq DR Daun JK Quality of Western Canadian Flaxseed. Canadian Grain Commission 2002 http://www.grainscanada.gc.ca/flax-lin/trend-tendance/qfc-qlc-eng.htm

[B42] Truksa M MacKenzie Samuel L Qiu X Molecular analysis of flax 2S storage protein conlinin and seed specific activity of its promoter Plant Physiology and Biochemistry 2003 41 141 147 10.1016/S0981-9428(02)00022-0

[B43] Ohlrogge JB Jaworski JG REGULATION OF FATTY ACID SYNTHESIS Annual Review of Plant Physiology and Plant Molecular Biology 1997 48 109 136 10.1146/annurev.arplant.48.1.109 15012259

[B44] Voelker T Kinney AJ VARIATIONS IN THE BIOSYNTHESIS OF SEED-STORAGE LIPIDS Annual Review of Plant Physiology and Plant Molecular Biology 2001 52 335 361 10.1146/annurev.arplant.52.1.335 11337402

[B45] Huang A Oleosins and Oil Bodies in Seeds and Other Organs Plant Physiol 1996 110 1055 1061 10.1104/pp.110.4.1055 8934621 PMC160879

[B46] Crowe AJ Abenes M Plant A Moloney MM The seed-specific transactivator, ABI3, induces oleosin gene expression Plant Science 2000 151 171 181 10.1016/S0168-9452(99)00214-9 10808073

[B47] Davin LB Lewis NG Dirigent Proteins and Dirigent Sites Explain the Mystery of Specificity of Radical Precursor Coupling in Lignan and Lignin Biosynthesis Plant Physiol 2000 123 453 462 10.1104/pp.123.2.453 10859176 PMC1539258

[B48] Ford JD Huang KS Wang HB Davin LB Lewis NG Biosynthetic Pathway to the Cancer Chemopreventive Secoisolariciresinol Diglucoside-Hydroxymethyl Glutaryl Ester-Linked Lignan Oligomers in Flax (Linum usitatissimum) Seed† Journal of Natural Products 2001 64 1388 1397 10.1021/np010367x 11720519

[B49] Oomah BD Giuseppe M Kenaschuk EO Flavonoid content of flaxseed. Influence of cultivar and environment Euphytica 1996 90 163 167 10.1007/BF00023854

[B50] Lepiniec L Debeaujon I Routaboul JM Baudry A Pourcel L Nesi N Caboche M GENETICS AND BIOCHEMISTRY OF SEED FLAVONOIDS Annual Review of Plant Biology 2006 57 405 430 10.1146/annurev.arplant.57.032905.105252 16669768

[B51] Xie DY Sharma SB Paiva NL Ferreira D Dixon RA Role of Anthocyanidin Reductase, Encoded by BANYULS in Plant Flavonoid Biosynthesis Science 2003 299 396 399 10.1126/science.1078540 12532018

[B52] Fedeniuk RW Biliaderis CG Composition and Physicochemical Properties of Linseed (Linum usitatissimum L.) Mucilage Journal of Agricultural and Food Chemistry 1994 42 240 247 10.1021/jf00038a003

[B53] Naran R Chen G Carpita NC Novel Rhamnogalacturonan I and Arabinoxylan Polysaccharides of Flax Seed Mucilage Plant Physiol 2008 148 132 141 10.1104/pp.108.123513 18667723 PMC2528086

[B54] Cui W Mazza G Biliaderis CG Chemical Structure, Molecular Size Distributions, and Rheological Properties of Flaxseed Gum Journal of Agricultural and Food Chemistry 1994 42 1891 1895 10.1021/jf00045a012

[B55] Western TL Young DS Dean GH Tan WL Samuels AL Haughn GW MUCILAGE-MODIFIED4 Encodes a Putative Pectin Biosynthetic Enzyme Developmentally Regulated by APETALA2, TRANSPARENT TESTA GLABRA1, and GLABRA2 in the Arabidopsis Seed Coat Plant Physiol 2004 134 296 306 10.1104/pp.103.035519 14701918 PMC316309

[B56] Dean GH Zheng H Tewari J Huang J Young DS Hwang YT Western TL Carpita NC McCann MC Mansfield SD The Arabidopsis MUM2 Gene Encodes a {beta}-Galactosidase Required for the Production of Seed Coat Mucilage with Correct Hydration Properties Plant Cell 2007 19 4007 4021 10.1105/tpc.107.050609 18165329 PMC2217648

[B57] Arsovski AA Popma TM Haughn GW Carpita NC McCann MC Western TL AtBXL1 Encodes a Bifunctional {beta}-D-Xylosidase/{alpha}-L-Arabinofuranosidase Required for Pectic Arabinan Modification in Arabidopsis Mucilage Secretory Cells Plant Physiol 2009 150 1219 1234 10.1104/pp.109.138388 19458117 PMC2705025

[B58] Harholt J Suttangkakul A Vibe Scheller H Biosynthesis of Pectin Plant Physiol 2010 153 384 395 10.1104/pp.110.156588 20427466 PMC2879803

[B59] Day A Addi M Kim W David H Bert F Mesnage P Rolando C Chabbert B Neutelings G Hawkins S ESTs from the Fibre-Bearing Stem Tissues of Flax (Linum usitatissimum L.): Expression Analyses of Sequences Related to Cell Wall Development Plant Biology 2005 7 23 32 10.1055/s-2004-830462 15666211

[B60] Fenart S Ndong YP Duarte J Riviere N Wilmer J van Wuytswinkel O Lucau A Cariou E Neutelings G Gutierrez L Development and validation of a flax (Linum usitatissimum L.) gene expression oligo microarray BMC Genomics 2010 11 592 10.1186/1471-2164-11-592 20964859 PMC3091737

[B61] Venglat SP Sawhney VK Benzylaminopurine induces phenocopies of floral meristem and organ identity mutants in wild-type *Arabidopsis *plants Planta 1996 198 480 487 10.1007/BF00620066 8717139

